# Persistent abnormal muscle response after microvascular decompression for hemifacial spasm

**DOI:** 10.1038/s41598-020-75742-x

**Published:** 2020-10-28

**Authors:** Li Xu, Wu Xu, Jing Wang, Yulong Chong, Weibang Liang, Chengrong Jiang

**Affiliations:** Department of Neurosurgery, Nanjing Drum Tower Hospital Clinical College of Nanjing Medical University, 321 Zhongshan Road, Nanjing, 210009 Jiangsu China

**Keywords:** Clinical trials, Neurovascular disorders

## Abstract

To explore the causes of persistent abnormal muscle response (AMR) after microvascular decompression (MVD) for hemifacial spasm (HFS) and the clinical outcomes of these patients. MVDs performed in Nanjing Drum Tower Hospital in 2017 were retrospectively studied, and 326 patients with HFS were classified into two groups based on whether AMR disappeared or persisted following MVD. The clinical features, treatment efficacy and postoperative complications were compared between the two groups. 305 patients with disappeared AMR after decompression were classified as Group A. In Group B, the 21 patients exhibited persistent AMR after successful MVD. The preoperative duration of symptoms in Group B was significantly longer than that in Group A (*P* < 0.001), and no significant difference was identified between the two groups in terms of gender, side, age and offending vessels (*P* > 0.05). The immediate postoperative cure rate of Group A (88.9%)was significantly higher than that in Group B (28.6%, *P* < 0.001), furthermore, the two groups were not different in the long-term outcome and the incidence of surgical complications (*P* > 0.05). The long preoperative duration of HFS patients may account for persistent AMR after successful decompression, and it is more likely for these patients to get delayed cured, the long-term outcomes showed no difference compared to those in patients with disappeared AMR after MVD.

## Introduction

Hemifacial spasm (HFS) refers to a cranial nerve disease characterized by involuntary contractions of muscles innervated by the ipsilateral facial nerve^1^. It could be secondary to compressive tumors or caused by demyelination, trauma, stroke, and Bell’s palsy^2,3^. For primary HFS, the most commonly proposed etiology is neurovascular compression (NVC) at the root exit zone (REZ) of the facial nerve, and nowadays, microvascular decompression (MVD) has now been suggested as an effective treatment^4,5^. Existing studies^6–8^ verified that abnormal muscle response (AMR) can help surgeons to identify the offending vessels, and its disappearance commonly indicates spasm free after decompression. However, not all the HFS patients showed AMR disappearance after successful decompression, AMR may be residual in some patients even after adequate decompression of offending vessels^1,9,10^. In the present study, the clinical features, intraoperative findings of the patients who received MVD for HFS in our institution in 2017 were retrospectively analyzed. The major aim was to delve into the cause of persistent AMR after MVD and the clinical outcomes of these patients.

## Materials and methods

### Research population

**Figure 1:**
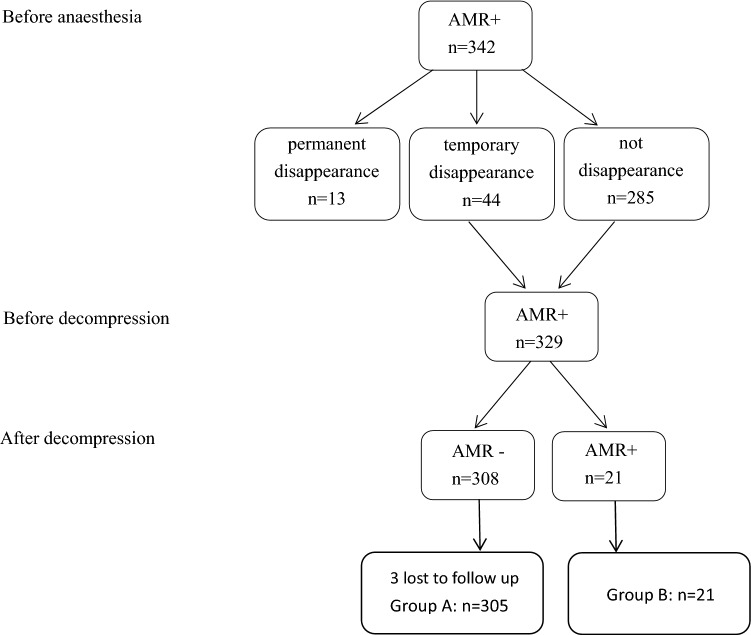
Number of patients and corresponding change of AMR.

The study was approved by the Ethical Committee of Nanjing Drum Tower Hospital, and written informed consent was obtained from each subject before the study. All methods were carried out in accordance with approved institutional guidelines and regulations. In this study, 342 patients having undergone MVD for HFS at our institution in 2017 were retrospectively studied. All of these patients were diagnosed with primary HFS, besides, the surgery was performed under the electrophysiological monitoring of AMR. 16 patients were excluded, including 13 patients exhibiting disappeared AMR before decompression and 3 who lost to follow up. Lastly, 326 patients were overall studied in this research. The patients fell into two groups based on whether AMR remained after successful decompression. Group A (305 cases): AMR showed a complete disappearance after decompression. Group B (21 cases): AMR was not eliminated though satisfactory decompression had achieved (Fig. 1).

### Decompression procedures and intraoperative AMR monitoring

With the patients in lateral decubitus position, the suboccipital retrosigmoid approach was performed. After identifying the edge of the sigmoid sinus, the dura mater and the arachnoids were thoroughly opened under the microscope. Then, the REZ of facial nerve was full exposured, and every suspected offending vessel was separated from the facial nerve root with shredded Teflon implants. If the AMR was eliminated after the offending vessel was transposed, this study considered that adequate decompression was achieved. If AMR was reported to be persistent or reappeared following satisfactory decompression, the conflict site should be reexamined to verify that no offending vessels were misidentified nor missed. Subsequently, microsurgical procedures should be completed.

All the patients were general anesthetized with propofol, remifentanil, combined tracheal intubation. And general anesthesia was induced by using a short-duration muscle relaxant, no more muscle relaxant was administered after anesthesia induction. The evoked potential system (Xltek Protektor32, Natus, USA) was exploited to monitor intraoperatively for AMR in our institution. A square-wave stimulation under 0.2 ms pulse width and 1 Hz frequency was employed. By stimulating the marginal mandibular branch of the facial nerve, AMR could be recorded from the orbicularis oculi and mentalis muscles. On the whole, a stable AMR could be recorded under a stimulation intensity of 5–20 mA. The AMR was evoked per 5 min before dual opening; after the dura mater was opened, the AMR was recorded continuously till the end of the operation^1^.

### Statistical analysis

The follow-up was carried out for all the patients by telephone communication or return visit in the period from 18 to 30 months (24.8 months on average), the outcomes and complications of the surgery were recorded. Statistical analyses were performed by using SPSS, version 22.0 (IBM, Armonk, NY). Data accorded with normal distribution and homogeneity of variance were compared by *t* test, and Chi-squared test, Fisher’s exact test were used to compare numerical variables. Statistical significance was defined at *P* < 0.05.

## Results

Among the 326 patients recruited in this study, 305 exhibited disappeared AMR after decompression, and they were classified as Group A. In this group, the mean age was 52.9 ± 6.85 years, 169 (55.4%) were female, and 172 of 305 (56.4%) developed left-sided symptoms; the mean duration before operation reached 23.4 ± 4.01 months. 271 (88.9%) patients were cured immediately. Moreover, delayed resolution^4^ (2 days to 18 months after surgery) was identified in 32 (10.5%) patients. 2 (0.7%) patients did not develop spasm free after receiving surgery. 14 (4.6%) cases of postoperative cranial nerve complications were observed, including 11 patients of facial paralysis, 1 of hoarseness and 3 of hearing loss. Complications were defined as permanent if they continued until the last follow-up. In this group, except for 1 patient developing permanent facial palsy, the other patients were recovered at the follow-up period. And, we observed 3 cases of recurrence in this group.

In Group B, AMR persisted in the 21 patients though adequate decompression had been achieved. 10 (47.6%) of the 21 patients were women, and the mean age was 55.3 ± 6.32 years. 8 (38.1%) patients were left-sided HFS, the mean duration before operation was 39.8 ± 9.63 months. After surgery, 6 (28.6%) patients achieved complete relief of HFS. There were 14 (66.7%) cases of delayed resolution, and the patients were completely cured 7 to 310 days after the surgery. 1 (4.8%) patients were not ameliorated till the end of follow up. There were 2 cases of transient facial palsy, no other cranial nerve complications were observed in this group. At the end of follow up,1 patient had a relapse. All the patients of the two groups had no deaths, strokes, cerebellar hematomas or infections.

After Analyzing the data with SPSS Statistics version 22.0, we found that the preoperative duration of symptoms in Group B was significantly longer than that in Group A (*P* < 0.001), and no significant difference was identified in terms of age, gender, affected side and offending vessels (*P* > 0.05) between the two groups (Table 1). Group A (88.9%) had a significantly higher immediate postoperative cure rate than Group B (28.6%, *P* < 0.001), whereas the two groups were not different in the long-term outcome (*P* = 0.068 > 0.05), demonstrating that patients in Group B were more likely to experience delayed resolution (Table 2). Furthermore, there was no statistically significant difference in postoperative and long-term complications between the two groups (*P* > 0.05) (Table 2).

**Table 1:** Clinical characters and offending vessels of two groups.

	Group A (n = 305)	Group B (n = 21)	*P* value
Gender (male/female)	136/169	11/10	0.490
Side (left/right)	172/133	8/13	0.103
Mean age (years)	52.9 ± 6.85	55.3 ± 6.32	0.120
Mean duration (months)	23.4 ± 4.01	39.8 ± 9.63	< 0.001
**Offending vessels**			
AICA	113 (37.0%)	8 (38.1%)	0.670
PICA	84 (27.5%)	5 (23.8%)	
AICA + PICA	27 (8.9%)	2 (9.5%)	
AICA + VA	41 (13.4%)	3 (14.3%)	
PICA + VA	32 (10.5%)	2 (9.5%)	
VA	6 (2.0%)	0	
AICA + PICA + VA	2 (0.7%)	1 (4.8%)

**Table 2:** Postoperative and long term outcomes and complications of two groups.

	Postoperative		Long-term	
	Group A	Group B	Group A	Group B
**Surgical outcomes**				
Cured	271 (88.9%)	6 (28.6%)	300 (98.4%)	19 (90.5%)
Non cured	34 (11.1%)	15 (71.4%)	5 (1.6%)	2 (9.5%)
*P* value	< 0.001		0.068	
**Complications**				
Yes	14 (4.6%)	2 (9.5%)	1 (0.3%)	0
No	291 (95.4%)	19 (90.5%)	304 (99.7%)	21 (100%)
*P* value	0.275		1	

## Discussion

Nowadays, microvascular decompression is recognized as an effective treatment for HFS. AMR, initially proposed by Moller and Jannetta in 1980s, is an abnormal electromyographic response characteristic of HFS patients^11^. It may help distinguish the disease from other facial movement disorders like Meige Syndrome, blepharospasm, neuromyotonia^12,13^. If AMR disappeared after the suspected offending vessel was moved or separated from the REZ of facial nerve during surgery, it could be regarded as the responsible vessel, and the patient would be more likely to achieve spasm free. Otherwise, the surgeon may misidentify or miss the exact offending vessels. Thus, AMR disappearance is considered as an indicator of adequate decompression. Moreover, previous studies^6,7,14,15^ proved that the disappearance of AMR after decompression was significantly associated with good outcomes.

However, not all the patients exhibited AMR disappearance after receiving successful decompression. Thirumala et al.^14^ reported that 40 (13.7%) of 293 HFS patients presented persistent AMR after surgery. Huang et al.^16^ studied 1138 patients with hemifacial spasm, and AMR did not disappeared in 72 (6.3%) cases 1 day after MVD, even after 1 year follow-up, AMR still could be evoked in 34 (3.0%) patients. According to existing studies^14,17–20^, the percentage of persistent AMR after MVD was from 3.1 to 25.6%. In present study, there were 21 (6.4%) patients who had underwent sufficient decompression, and every vessel relevant to the facial nerve was moved away and no neurovascular compression could be identified. However, AMR did not disappear till the end of operation. As suggested by some existing studies, the regeneration of the demyelination of facial nerve or the stabilization of the facial motor nucleus cost some time, which might account for persistent AMR after adequate decompression^21,22^. By comparing the clinical features of the two groups, this study reported that the preoperative duration of the patients exhibiting persistent AMR was significantly longer that of AMR-disappear patients. Thus, we believe that there was a significant correlation between AMR changes and preoperative duration of HFS.

For HFS patients, AMR disappearance after decompression usually correlates with better outcomes^8,14,18^, but persistent AMR does not always mean a poor outcome. Kong et al.^21^ reported 33 of 263 HFS patients showed persistent AMR after MVD, and 17 cases (51.5%) experienced spasm free 1 week later, at the 1-year follow-up examination, 22 cases (66.7%) suggested complete relief. In Some scholars’ research, all the patients exhibiting persistent AMR after decompression were free of spasms during the follow-up period^22,23^. In the present study, only 6 (28.6%) of the 21 patients had spasm free immediately after MVD. Compared with Group A, the immediate cure rate was significantly lower. But at the end of follow up, no significant difference was seen in long term outcomes. Therefore, we suggested that the patients exhibiting persistent AMR after decompression had a greater chance to get delayed resolution.

The concept of delayed resolution of HFS have been already accepted, and the delayed cure rate was about 2.9–50.3%^4,24^. Though the exact mechanism of delayed resolution is debated, it has been demonstrated that the delayed resolution time was correlated with preoperative symptom duration^5,25^. According to previous studies^4,5,16,25^, we suggested that for the patients with a long preoperative duration, even though adequate decompression has been achieved, the regeneration of the injured facial nerve or the stabilization of the facial motor nucleus still takes more time, there is a higher probability to present persistent AMR, and the patients were more likely to achieve delayed resolution. Moreover, the exact reason of delayed resolution and its relationship to AMR require in-depth study.

In this study, we observed 2 cases of facial paralysis in Group B, while in Group A, postoperative complications were observed in 14 patients. Statistically, no significant difference was identified in the postoperative and long term complications of the two groups (*P* > 0.05). We believe that if AMR disappeared after the offending vessel is transposed, the procedure should be finished, if AMR remains persist after decompression, the REZ of facial nerve should be reexamined rigorously, as an attempt to avoid misidentify or miss the exact offending vessels. If adequate decompression is confirmed to be achieved, the operations should be finished, in case of more frequent complications result from unnecessary manipulations.

Today, AMR monitoring is extensively applied in MVD for HFS patients, and considerable researches suggested that AMR disappearance after decompression could significantly assess good outcome after MVD^19–22^. However, it should be stressed that the key to successful MVD is sufficient decompression, persistent AMR does not definitely predict a poor outcome. On the other hand, the sensitivity and accuracy of AMR may be affected by muscle relaxant and some Surgical instruments^26,27^. For this reason, there were some limitations of intraoperative AMR monitoring. It should not be regarded as an absolute standard to predict clinical outcomes.

## Conclusion

The present retrospective study focused on the AMR changes of 326 HFS patients who received MVD, especially the 21 patients exhibiting persistent AMR after successful decompression. We believe that preoperative duration is correlated with AMR persistence after decompression, and these patients were more likely to experience delayed resolution. Whether AMR disappears after decompression is not correlated with the long term outcomes and surgical complications. For patients with persistent AMR, we suggest that surgeons should not perform any further exploration once sufficient decompression has been achieved.

## References

[1] Jiang C, Xu W, Dai Y, Lu T, Jin W, Liang W (2017). Early permanent disappearance of abnormal muscle response during microvascular decompression for hemifacial spasm: a retrospective clinical study. Neurosurg. Rev..

[2] Ruiz-Juretschke F, Vargas A, González-Rodrigalvarez R, Garcia-Leal R (2015). Hemifacial spasm caused by a cerebellopontine angle arachnoid cyst. Case report and literature review. Neurocirugia (Astur).

[3] Ghali MGZ, Srinivasan VM, Viswanathan A (2018). Microvascular decompression for hemifacial spasm. Int. Ophthalmol. Clin..

[4] Jo KW, Kong DS, Park K (2013). Microvascular decompression for hemifacial spasm: long-term outcome and prognostic factors, with emphasis on delayed cure. Neurosurg. Rev..

[5] Hyun SJ, Kong DS, Park K (2010). Microvascular decompression for treating hemifacial spasm: lessons learned from a prospective study of 1,174 operations. Neurosurg. Rev..

[6] Kim CH, Kong DS, Lee JA, Park K (2010). The potential value of the disappearance of the lateral spread response during microvascular decompression for predicting the clinical outcome of hemifacial spasms: a prospective study. Neurosurgery.

[7] Sekula RF, Bhatia S, Frederickson AM (2009). Utility of intraoperative electromyography in microvascular decompression for hemifacial spasm: a meta-analysis. Neurosurg. Focus.

[8] Fukuda M, Oishi M, Takao T, Hiraishi T, Sato Y, Fujii Y (2012). Monitoring of abnormal muscle response and facial motor evoked potential during microvascular decompression for hemifacial spasm. Surg. Neurol. Int..

[9] Hirono S, Yamakami I, Sato M (2014). Continuous intraoperative monitoring of abnormal muscle response in microvascular decompression for hemifacial spasm; a real-time navigator for complete relief. Neurosurg. Rev..

[10] Li J, Zhang Y, Zhu H, Li Y (2012). Prognostic value of intra-operative abnormal muscle response monitoring during microvascular decompression for long-term outcome of hemifacial spasm. J. Clin. Neurosci..

[11] Møller AR (1991). Interaction between the blink reflex and the abnormal muscle response in patients with hemifacial spasm: results of intraoperative recordings. J. Neurol. Sci..

[12] Valls-Solé J (2002). Facial palsy, postparalytic facial syndrome, and hemifacial spasm. Mov. Disord..

[13] Huang C, Miao S, Chu H, Muheremu A, Wu J, Zhou R, Zuo H, Ma Y (2016). Application of electrophysiological methods and magnetic resonance tomographic angiography in the differentiation between hemifacial spasm and Meige syndrome. Neurol. Sci..

[14] Thirumala PD, Shah AC, Nikonow TN (2011). Microvascular decompression for hemifacial spasm: evaluating outcome prognosticators including the value of intraoperative lateral spread response monitoring and clinical characteristics in 293 patients. J. Clin. Neurophysiol..

[15] von Eckardstein K, Harper C, Castner M, Link M (2014). The significance of intraoperative electromyographic "lateral spread" in predicting outcome of microvascular decompression for hemifacial spasm. J. Neurol. Surg. B Skull Base.

[16] Huang C, Miao S, Chu H, Dai C, Wu J, Wang J, Zuo H, Ma Y (2017). An optimized abnormal muscle response recording method for intraoperative monitoring of hemifacial spasm and its long-term prognostic value. Int. J. Surg..

[17] Lee S, Park SK, Lee JA, Joo BE, Kong DS, Seo DW, Park K (2018). A new method for monitoring abnormal muscle response in hemifacial spasm: A prospective study. Clin. Neurophysiol..

[18] Huang BR, Chang CN, Hsu JC (2009). Intraoperative electrophysiological monitoring in microvascular decompression for hemifacial spasm. J. Clin. Neurosci..

[19] Wei Y, Yang W, Zhao W, Pu C, Li N, Cai Y, Shang H (2018). Microvascular decompression for hemifacial spasm: can intraoperative lateral spread response monitoring improve surgical efficacy?. J. Neurosurg..

[20] Lee SH, Park BJ, Shin HS, Park CK, Rhee BA, Lim YJ (2017). Prognostic ability of intraoperative electromyographic monitoring during microvascular decompression for hemifacial spasm to predict lateral spread response outcome. J. Neurosurg..

[21] Kong DS, Park K, Shin BG, Lee JA, Eum DO (2007). Prognostic value of the lateral spread response for intraoperative electromyography monitoring of the facial musculature during microvascular decompression for hemifacial spasm. J. Neurosurg..

[22] Hatem J, Sindou M, Vial C (2001). Intraoperative monitoring of facial EMG responses during microvascular decompression for hemifacial spasm. Prognostic value for long-term outcome: a study in a 33-patient series. Br. J. Neurosurg..

[23] Kiya N, Bannur U, Yamauchi A, Yoshida K, Kato Y, Kanno T (2001). Monitoring of facial evoked EMG for hemifacial spasm: a critical analysis of its prognostic value. Acta Neurochir. (Wien).

[24] Goto Y, Matsushima T, Natori Y, Inamura T, Tobimatsu S (2002). Delayed effects of the microvascular decompression on hemifacial spasm: a retrospective study of 131 consecutive operated cases. Neurol. Res..

[25] Shin JC, Chung UH, Kim YC, Park CI (1997). Prospective study of microvascular decompression in hemifacial spasm. Neurosurgery.

[26] Cai YR, Xu J, Chen LH, Chi FL (2009). Electromyographic monitoring of facial nerve under different levels of neuromuscular blockade during middle ear microsurgery. Chin. Med. J. (Engl.).

[27] Fang Y, Zhang H, Liu W, Li Y (2012). A comparison of three induction regimens using succinylcholine, vecuronium, or no muscle relaxant: impact on the intraoperative monitoring of the lateral spread response in hemifacial spasm surgery: study protocol for a randomised controlled trial. Trials.

